# Selective localization of IgG from cerebrospinal fluid to brain parenchyma

**DOI:** 10.1186/s12974-018-1159-8

**Published:** 2018-04-17

**Authors:** Marlene Thorsen Mørch, Sofie Forsberg Sørensen, Reza Khorooshi, Nasrin Asgari, Trevor Owens

**Affiliations:** 1 Neurobiology, Institute of Molecular Medicine, University of Southern Denmark, JB. Winsloewsvej 25, 5000 Odense, Denmark; 2 Department of Neurology, Slagelse Hospital, Institute of Regional Health Research, Slagelse, Denmark

**Keywords:** Immunoglobulin-G, Cerebrospinal fluid, Neuromyelitis optica spectrum disorders, Pathology, Deposition

## Abstract

**Background:**

Encounter of autoantibodies with specific antigens can lead to hypersensitivity reactions and pathology. In multiple sclerosis and neuromyelitis optica spectrum disease (NMOSD), immunoglobulin-G (IgG) deposition has been observed in pathological lesions in the central nervous system. The paradigmatic autoantibodies in NMOSD are specific for the water channel aquaporin-4, localized to astrocytic end-feet at the blood-brain barrier and ependymal cells at the cerebrospinal fluid-brain barrier. We have previously observed that IgG antibodies from NMO patients (NMO-IgG) access brain parenchyma from the cerebrospinal fluid and induce subpial and periventricular NMO-like lesions and blood-brain barrier breakdown, in a complement-dependent manner.

**Objective:**

To investigate how IgG trafficking from cerebrospinal fluid to brain parenchyma can be influenced by injury.

**Methods:**

IgG from healthy donors was intrathecally injected into the cerebrospinal fluid via cisterna magna at 1, 2, 4, or 7 days after a distal stereotactic sterile needle insertion to the striatum.

**Results:**

Antibody deposition, detected by staining for human IgG, peaked 1 day after the intrathecal injection and was selectively seen close to the needle insertion. When NMO-IgG was intrathecally injected, we observed complement-dependent NMO-like pathology (loss of aquaporin-4 and glial fibrillary acidic protein) proximal to the insertion site, with similar kinetics. A fluorescent tracer did not show the same distribution indicating IgG-selective localization.

**Conclusion:**

These findings suggest that IgG from cerebrospinal fluid localize selectively in brain parenchyma at the site of injury and pathogenic NMO-IgG induce astrocyte pathology at the same location.

## Introduction

Inflammatory demyelinating diseases of the central nervous system (CNS) result in destruction of the myelin sheath and nerve fibers, which results in disability [1]. Whereas multiple sclerosis (MS) is considered a disease with myelin-directed immunity, in neuromyelitis optica spectrum disease (NMOSD), astrocytes are the primary target of the immune response [1, 2]. In NMOSD, the target antigen is identified as aquaporin-4 (AQP4) [3]. In the CNS, AQP4 is expressed by astrocytes and localized to astrocytic end-feet at the blood-brain barrier (BBB) and ependymal cells at the cerebrospinal fluid (CSF)-brain barrier [4, 5]. NMOSD lesions are predominantly located at the optic nerve or spinal cord, but specific brain MRI abnormalities including brainstem lesions are being increasingly recognized [6, 7]. Elevated titers of immunoglobulin-G (IgG) are seen in CSF in MS [8] and during relapse in NMOSD [9]. In NMOSD, anti-AQP4 antibodies in the CSF correlate with astrocyte damage, the primary pathologic process [10]. Deposition of IgG and complement is observed in NMOSD pathological lesions [11], as well as in a subset of MS patients with pattern II lesions [12]. There is a need for better understanding of the clinical relevance of antibodies in CSF and how they access the parenchyma.

Several animal models have been developed to examine the mechanism behind NMO-like pathology [13–16]. We have demonstrated that antibodies from NMO patients (NMO-IgG), with human complement that was intrathecally injected into the CSF via cisterna magna, induced NMO-like lesions with loss of AQP4 and glial fibrillary acidic protein (GFAP). These lesions co-localized with deposition of human IgG and activated complement. Notably, lesions were widespread in the brain parenchyma, and lesion location varied from mouse to mouse [15]. We showed that antibodies from the CSF were deposited via a paravascular route in subarachnoid and subpial space and in the periventricular region. Perivascular deposition was specific for NMO-IgG [14]. We further observed that NMO-IgG induced local complement-dependent loss of BBB integrity at specific but variable sites within brain parenchyma [14].

There is broad consensus that AQP4/NMO-IgG initially enters the brain via BBB-deficient sites such as area postrema, where access to CSF occurs [17]. NMO-IgG from the CSF gains access to the brain parenchyma and induces NMO-like lesions [15], with BBB disruption, via a specific IgG distribution pattern [14]. There is a need for further studies of pathogenic mechanism of IgGs and a better comprehension of what influences distribution of CSF-derived IgG in the parenchyma, to help understand how this site specificity is determined. Whether and how fluid flow occurs from CSF to CNS parenchyma is currently under debate [18–20]. Because little is known about what determines CSF IgG distribution in parenchyma, we examined if external factors, such as a sterile needle insertion, could influence the distribution.

## Materials and methods

### Animals

Adult female C57Bl/6J mice (Taconic, Lille Skensved, Denmark) were housed in The Biomedical Laboratory, University of Southern Denmark. Forty-six mice were included for examination of IgG or dextran distribution while four mice, one sample per brain hemisphere, were included for the gene expression analysis.

### Sterile needle insertion

Mice were anesthetized by isoflurane inhalation. Stereotactic coordinates were 2 mm lateral from bregma and 0.2 mm anterior. A 30-gauge needle attached to a 50-μl Hamilton syringe was inserted 3.5 mm in the right hemisphere into the striatum. Ten microliters of PBS was infused (2 μl/min) following needle insertion. After injury, mice received Temgesic (RB Pharmaceuticals Limited, Berkshire, UK) for pain relief and isotonic NaCl subcutaneously to prevent dehydration.

### Intrathecal delivery

One, 2, 4, or 7 days after the sterile needle insertion, a 30-gauge needle (bent at 55°, 2–2.5 mm from the tip) attached to a 50-μl Hamilton syringe was inserted between the skull and the cervical vertebra into the intrathecal space of the cisterna magna. Three hundred micrograms of human antibodies either NMO-IgG or normal-IgG [15] and 288 μg complement protein isolated from healthy donors or 125 μg Dextran, fluoresceinated, 40,000 MW (Molecular Probes, Life Technologies, Eugene, OR, USA) in a total volume of 10 μl. Anesthesia and analgesia were as described above.

### Tissue processing

Mice were euthanized by an overdose of sodium pentobarbital and subsequently perfused transcardially with ice-cold PBS (10 ml) followed by 4% paraformaldehyde in PBS (20 ml). After removal, brains were post-fixed in 4% PFA, immersed in 30% sucrose in PBS for up to 2 weeks at 4 °C, then frozen in cryostat embedding medium (Killik, Milano, Italy) by immersion in 2-methylbutane (Sigma-Aldrich, Denmark) in liquid nitrogen. For examination of dextran diffusion, whole cerebra were cut serially into 30-μm coronal sections on a Micron HM 550 cryostat (Microm International GmbH, Walldorf, Germany). For examination of IgG diffusion, 16-μm sagittal or coronal serial sections through the lesion were cut on a Micron HM 550 cryostat. For examination of NMOSD-like pathology (loss of AQP4 and GFAP, deposition of IgG and C9neo, and BBB disruption), sagittal serial sections (50, 30, and 16 μm) were cut through the lesions. For examination of gene expression levels, mice were perfused with PBS, and a tissue block that included the needle insertion was stored in TRIzol reagent (Invitrogen-Molecular Probes, Eugene, OR, USA). For examination of BBB disruption, mice were intravenously injected with 2 mg horseradish peroxide (HRP) 15 min before perfusion.

### Immunohistochemistry

CNS tissue was evaluated for deposition of human IgG and C9neo, loss of AQP4, and GFAP staining.

Sections were dried then washed in PBS followed by three times wash in PBS containing 0.2% Triton X-100 (Sigma-Aldrich, St. Louis, MO, USA) (PBST). Sections were then blocked for endogenous peroxidases by immersion in methanol containing 0.2% H_2_O_2_ (Sigma-Aldrich, St. Louis, MO, USA) for 30 min at room temperature. Sections were thereafter washed in PBST three times. Unless otherwise stated, washes were carried out three times and all incubations were at room temperature. After washing, sections were blocked for non-specific staining with 3% bovine serum albumin (Sigma-Aldrich, St. Louis, MO, USA) in PBST for 30 min. Sections were incubated for 1 h with primary antibody (rabbit anti-AQP-4 (1:400) (Alomone Labs Ltd., Jerusalem, Israel), rabbit anti-GFAP (1:1000) (DAKO Denmark A/S, Glostrup, Denmark), rabbit anti-human IgG (1:200) (Abcam, Cambridge, UK), rabbit anti-C9neo (1:100) (Abcam, Cambridge, UK)). To verify antibody specificity, sections were incubated with corresponding concentrations of rabbit immunoglobulin fraction (DAKO Denmark A/S, Glostrup, Denmark). Sections were then washed in PBST and incubated for 1 h with secondary antibody (biotinylated goat anti-rabbit IgG (1:2) (Abcam, Cambridge, UK)). After washing in PBST, sections were incubated for 1 h with streptavidin-horseradish peroxide (1:200, GE Healthcare, Little Chalfont, Buckinghamshire, UK). Afterwards, sections were washed in PBS and developed by adding 3,3′-diaminobenzidine (DAB, 0.5 mg/ml) (Sigma-Aldrich, St. Louis, MO, USA) and H_2_O_2_ (0.033%) (Sigma-Aldrich, St. Louis, MO, USA) for 2 min and washed twice in PBS. Finally, sections were dehydrated using increasing concentrations of ethanol, cleared in xylene, and mounted using DPX mounting medium (Merck KGaA, Darmstadt, Germany).

IgG deposition, BBB disruption, and loss of AQP4 staining were analyzed on full series (between 12 to 30 sections), while GFAP loss and deposition of C9neo were examined on corresponding sections. Images were acquired using an Olympus BX51 microscope with an Olympus DP73 camera (Olympus, Ballerup, Denmark) and analyzed using the free software Fiji.

### Immunofluorescence staining

Dextran diffusion was investigated by co-staining for GFAP to identify the needle insertion. Slides with brain sections were dried and washed once in PBS and then three times in PBST. Sections were blocked for non-specific staining as above then incubated for 1 h with anti-GFAP (1:1000, Cy3) (Sigma-Aldrich, St. Louis, MO, USA). Slides were then washed once in PBS, then 5 min in PBS containing 300 nM 4,6-diamidino-2-phenylindole (Invitrogen) to stain nuclei, then washed in PBS and mounted using gelvatol [21].

Dextran diffusion was analyzed on a full series (approximately 40 sections). Images were acquired using an Olympus BX51 microscope with an Olympus DP73 camera and analyzed using Adobe Photoshop CS3 version 10.0.

### RNA extraction, reverse transcription, and quantitative reverse transcriptase/real-time PCR

RNA was extracted using TRIzol reagent (Invitrogen-Molecular Probes, Eugene, OR, USA) in accordance with the manufacturer’s protocol. One microgram of total RNA was reverse transcribed using M-MLV reverse transcriptase (Invitrogen) according to the manufacturer’s protocol. Primer and probe sequences were as follows: angiotensinogen (AGT) (forward TGAACAACATTGGTGACACCAA, reverse CTGCTTTGAGTTCGAGGAGGAT, probe TGGGAGAGGTTCTCAATAG MGB), angiotensin-converting enzyme (ACE) (forward CCTCTGCCTGGGACTTCTACA, reverse CGTGACCCGTGTGCATTG, probe AAGGACTTCCGGATTAA MGB), renin (forward GCACCTTCAGTCTCCCAACAC, reverse CCCGGACAGAAGGCATTTT, probe CTTTGAACGAATCCCGC MGB), IFNγ (forward CATTGAAAGCCTAGAAAGTCTGAATAAC, reverse TGGCTCTGCAGGATTTTCATG, probe TCACCATCCTTTTGCCAGTTCCTCCAG MGB), interleukin (IL)-4 (forward ACAGGAGAAGGGACGCCAT, reverse GAAGCCCTACAGACGAGCTCA, probe TCCTCACAGCAACGAAGAACACCACA MGB), IL-10 (forward GGTTGCCAAGCCTTATCGGA, reverse ACCTGCTCCACTGCCTTGCT, probe TGAGGCGCTGTCATCGATTTCTCCC MGB), TGFβ (forward TGACGTCACTGGAGTTGTACGG, reverse GGTTCATGTCATGGATGGTGC, probe TTCAGCGCTCACTGCTCTTGTGACAG MGB), TNFα (forward CCAAATGGCCTCCCTCTCAT, reverse TCCTCCACTTGGTGGTTTGC, probe CTCACACTCAGATCAT MGB), and iNOS (gene expression assay kit from TaqMan, Applied Biosystems Inc., Foster City, CA, USA). Samples were run as triplicates on an ABI Prism 7300 Sequence Detection System (Applied Biosystems Inc.). Results were expressed relative to 18S rRNA (2ΔCT method) as endogenous control (TaqMan® Ribosomal RNA control reagents kit; Applied Biosystems Inc). CDNA was diluted 1:1000 for 18S rRNA analysis.

### Statistics

Data was analyzed by nonparametric Mann–Whitney *t* test using GraphPad Prism version 4 (GraphPad Software Inc., San Diego, CA, USA). Data are presented as mean ± SEM. Values of *p* < 0.05 were considered statistically significant.

## Results

### Needle insertion into the brain parenchyma influences IgG trafficking

**Fig. 1:**
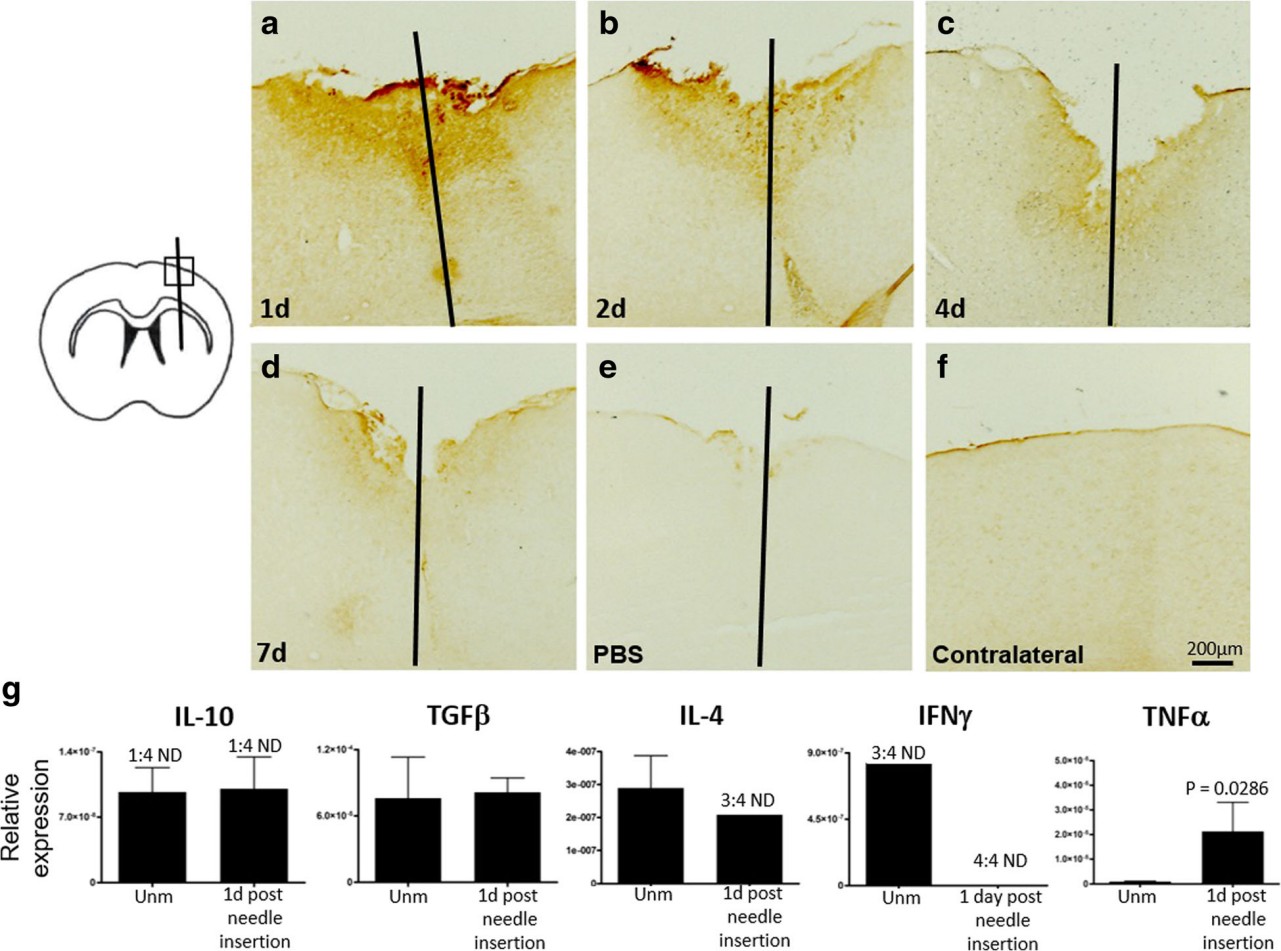
IgG trafficking is influenced by needle insertion. **a**–**f** Representative histological micrographs of frontal brain sections from mice showing IgG distribution in and around the needle track following intrathecal injection of normal-IgG 1, 2, 3, or 7 days after sterile needle insertion. Sections show higher deposition of IgG 1 day post needle insertion compared to later time points. **g** Comparison of relative expression of inflammation-associated cytokines (IL-10, TGFβ, IL-4, IFNγ, and TNFα) between the cortex and striatum from mice 1 day post needle insertion and unmanipulated (unm) mice. Data were analyzed by two-tailed nonparametric Student’s *t* test followed by the Mann–Whitney test. Results are presented as means ± SEM. Bar 200 μm

Groups of C57BL/6 mice received normal-human-IgG into the CSF by intrathecal injection 1, 2, 4, or 7 days post stereotactic insertion of a sterile needle to striatum. Histopathological analysis showed significant deposition of human IgG in the ipsilateral hemisphere compared to the corresponding area in the contralateral and a lack of staining following intrathecal injection of PBS (Fig. 1a–f). IgG deposition was localized to the area immediately proximal to needle insertion. Controls for non-specific staining were negative (not shown). Immunohistochemical analysis revealed that IgG deposition in the area around the needle insertion was strongest at 1 day (Fig. 1a) and was gradually reduced at 2, 4, and 7 days after the needle insertion (Fig. 1b–d). Hematoxylin and eosin staining at the time point for the strongest IgG deposition revealed only few infiltrating cells in the area where IgG was deposited (not shown). Among inflammation-associated cytokines (IFNγ, IL-4, IL-10, TGFβ, TNFα), only TNFα showed a significant upregulation by RT-qPCR (Fig. 1g).

### Distinct patterns of IgG versus dextran distribution

**Fig. 2:**
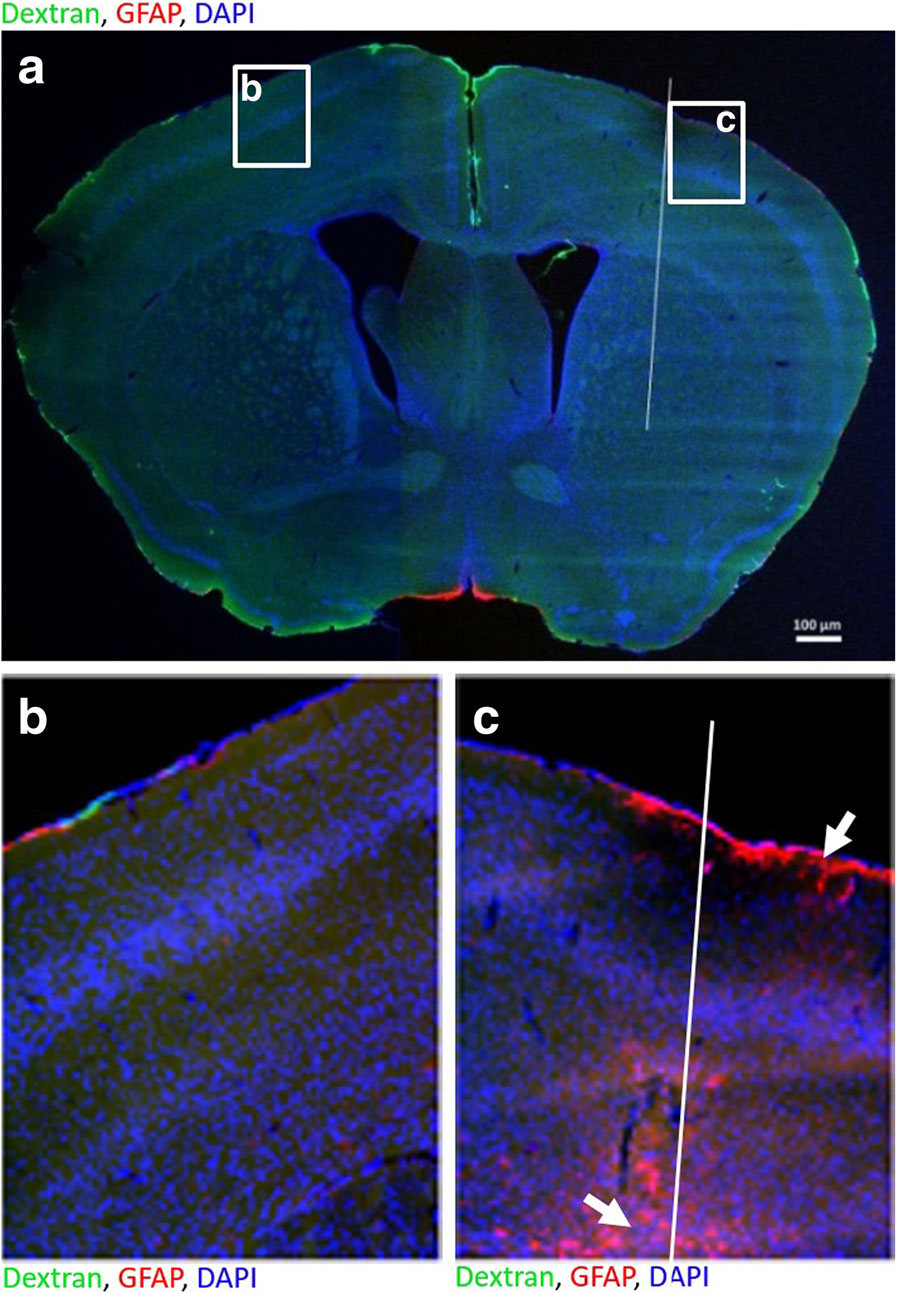
Dextran does not deposit at needle insertion. **a** Representative frontal brain section of a mouse intrathecally injected with fluoresceinated dextran (40,000 MW) 1 day post sterile needle insertion. The section was co-stained anti-GFAP (red). Dextran was not observed in and around the needle track. **b** and **c** show higher magnification of the contralateral and ipsilateral hemispheres, respectively. GFAP staining (red) was used to identify the needle track (white arrow). Bar 100 μm

To examine whether IgG simply followed CSF and interstitial fluid (ISF) flow, fluorescein-conjugated dextran (40,000 MW) was injected intrathecally to mice. We observed a distribution pattern similar to that described by Iliff et al. [18] (not shown). We then examined the effect of a sterile needle insertion to the striatum. No change in distribution of dextran was observed when it was intrathecally injected into mice 1 day after sterile needle insertion (Fig. 2a). Importantly, we did not observe dextran deposition in the area around the needle insertion (Fig. 2c), although we observed increased GFAP staining at this site (Fig. 2c, white arrow). This indicates that altered CSF/ISF flow was not directly responsible for the pattern of IgG deposition seen following needle insertion. Analysis of expression levels of genes associated with the renin-angiotensin system and with inducible nitric oxide synthase 1 day post needle insertion also showed no change (not shown).

### Pathogenic IgG induces NMOSD-like pathology

**Fig. 3:**
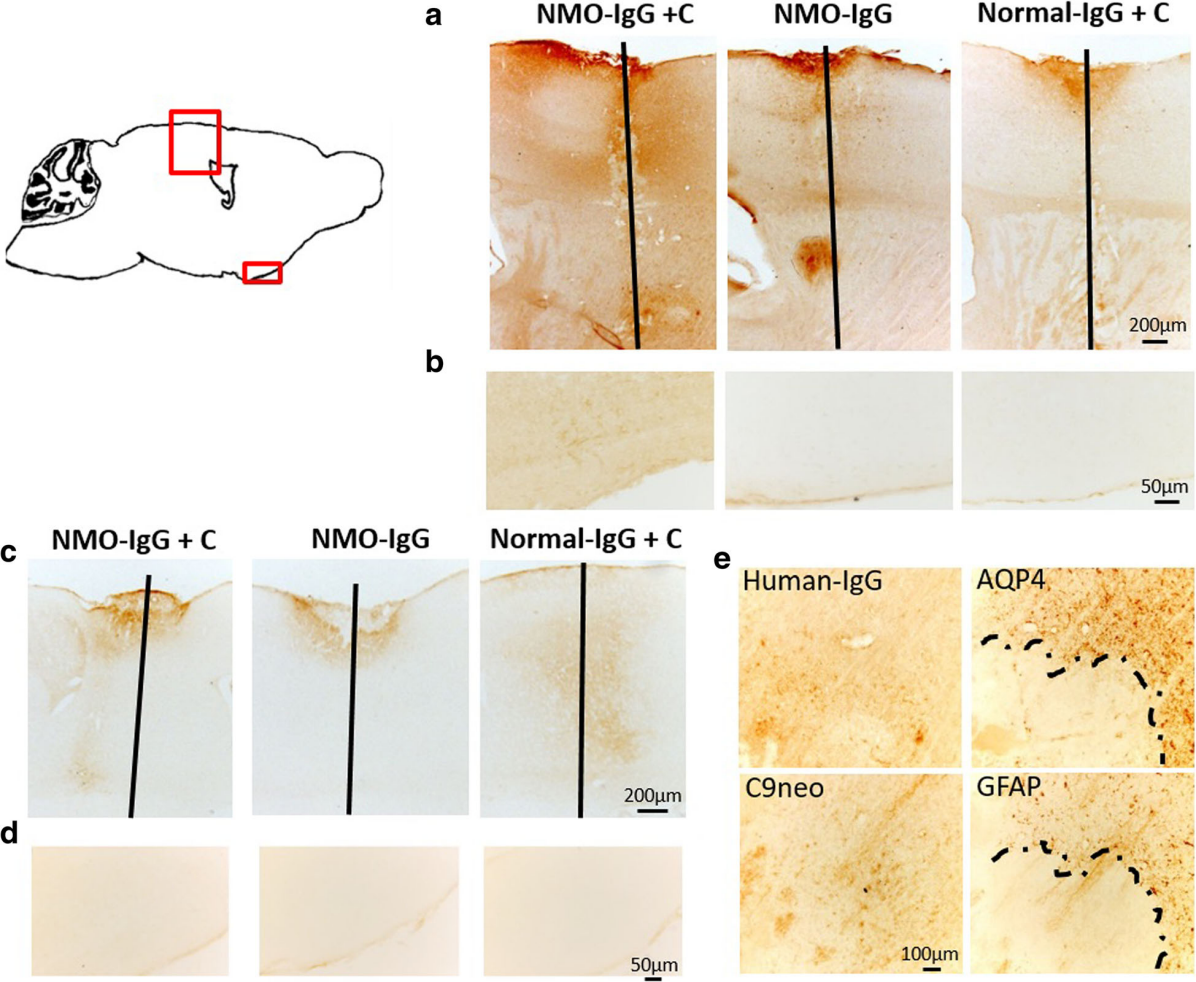
NMOSD-like pathology at the needle insertion. **a**, **b** Representative sagittal brain sections from mice intrathecally injected with NMO-IgG and complement, NMO-IgG alone, or normal-IgG with complement 1 day post needle insertion. **a** Micrograph showing IgG deposition in and around the needle track 1 day after intrathecal injection. **b** Low-level deposition of IgG away from the needle track 1 day after intrathecal injection. **c**, **d** Representative sagittal brain sections from mice intrathecally injected with NMO-IgG and complement, NMO-IgG alone, or normal-IgG and complement 1 day post needle insertion. **c** Micrograph showing IgG deposition in and around the needle track 3 days after intrathecal injection. **d** Lack of low-level deposition of IgG away from the needle track 3 days after intrathecal injection. **e** NMO-like pathology in mice 3 days after intrathecal injection. Mice received NMO-IgG and complement by intrathecal injection 1 day post needle insertion. Pathology was identified by loss of AQP4 and GFAP staining (brown) together with deposition of IgG and activated complement (C9neo) (brown). Bars 200 μm (**a**, **c**), 100 μm (**e**), and 50 μm (**b**, **d**)

We asked whether intrathecally injected pathological human antibodies would result in NMOSD-like pathology at the insertion site. Mice received NMO-IgG together with complement by intrathecal injection, or as controls NMO-IgG alone or normal-IgG with complement, 1 day post needle insertion. All groups showed significant deposition of human IgG at the insertion site in the ipsilateral hemisphere 1 day after the intrathecal injection (Fig. 3a). A low level of IgG deposition was detected at higher magnification throughout the areas adjacent to the interface with the CSF, possibly indicating passive diffusion of IgG from CSF to the parenchyma. This diffusion of IgG was strongest in mice that received NMO-IgG and complement, although was also detected in mice receiving NMO-IgG or normal-IgG + C (Fig. 3b). This low-level deposition did not result in noticeable loss of AQP4 or GFAP staining (not shown). Analysis of IgG deposition 3 days after intrathecal injection revealed that it was still significant at the insertion site for both NMO-IgG + complement and for controls (Fig. 3c). However, the low-level IgG diffusion seen 1 day after intrathecal injection was now absent (Fig. 3d). Furthermore, 1 day post intrathecal injection complement-dependent NMOSD-like pathology was observed at the site of sterile needle insertion in mice that received NMO-IgG and complement (3/3). Pathology was identified by loss of AQP4 and GFAP staining on astrocytes together with deposition of human-IgG and activated complement (C9neo) (Fig. 3e). One mouse that received NMO-IgG alone by intrathecal injection (1/4) showed loss of AQP4 and GFAP staining together with deposition of IgG and C9neo, suggesting unusual fixation of endogenous complement in this case (not shown). We also assessed BBB breakdown by intravenous injection of HRP. As expected, diffusion of HRP was observed associated to the needle track (not shown) and in association to NMO-like pathology (not shown).

## Discussion

In this study, we have shown that independent of pathogenicity and complement, a sterile needle insertion directs deposition of CSF-derived IgG in the brain parenchyma. Time intervals between needle insertion and intrathecal injection of human IgG were shown to be crucial to the extent of IgG deposition, deposition peaking transiently 1 day after needle insertion. We observed complement-dependent astrocyte pathology at the needle insertion site induced by intrathecally delivered NMO-IgG and complement, identified as loss of staining for the astrocytic markers AQP4 and GFAP together with deposition of IgG and activated complement. Consistent with our previous study [14], we observed HRP leakage from blood to parenchyma in and close to the needle track and together with astrocytic pathology. Cellular infiltration was not marked. Importantly, intrathecally delivered dextran did not show the same localization. Expression levels of cytokines associated with inflammation were not affected by needle insertion, except for TNFα which was significantly upregulated. TNFα has previously been shown to be upregulated after injury in both adult and neonatal mouse brain [22].

Detection of AQP4-IgG in CSF has been reported in NMOSD, and disease activity has been linked to high levels of AQP4-IgG in CSF and serum [9, 10, 23]. Furthermore, levels of AQP4-IgG in CSF strongly correlate with astrocyte damage, as reflected in elevated levels of soluble GFAP in CSF during NMO relapse [10, 24]. We have previously reported that NMO-IgG and complement injected intrathecally deposited in three patterns. We observed deposition in leptomeninges, subarachnoid space, and the subpial spaces where vessels penetrate the brain parenchyma [14]. The previously observed leptomeningeal deposition of NMO-IgG is in line with the observation that contrast enhancement of the leptomeningeal structures, indicating disruption of the leptomeningeal blood-barrier, has been observed in AQP4-IgG-positive patients during clinical attacks [25]. The observed distribution pattern of IgG was similar for normal-IgG and NMO-IgG. We also observed deposition in the periventricular region for both normal- and NMO-IgG. Interestingly, we observed perivascular deposition specifically for NMO-IgG and complement at brain parenchymal vessels distal from the site of parenchymal entry of pial vessels. This perivascular deposition was associated with astrocyte pathology and together with blood-borne HRP leakage into the parenchyma indicated that NMO-IgG in CSF has potential widespread distribution within the brain via a paravascular route to exert pathologic effects [14]. In this study, we also observed HRP leakage together with astrocyte pathology. We propose that IgG intrathecally injected after needle insertion can distribute from CSF to parenchyma via two different routes. One route would be via the above identified paravascular route, while the other route would be diffusion to parenchyma via the needle track.

It has been postulated that CSF flow is cardiac cycle-dependent and not unidirectional [19, 26, 27]. In principle, change in blood flow might affect the CSF flow, and a change in CSF flow could explain why antibodies deposited at the insertion site. However, examination of expression levels of iNOS and elements of the renin-angiotension system that are implicated in regulation of blood flow in the brain showed no change. Furthermore, intrathecal injection of a fluorescent dextran tracer showed no change in distribution between naïve mice and mice 1 day post needle insertion, indicating that CSF/ISF flow was not noticeably affected by the needle insertion. Increased AQP4 and GFAP immunoreactivities around the lesions were indeed observed, as also seen in clinical and experimental studies with or without needle insertion [11, 13]. However, this trauma-induced increased immunoreactivity of AQP4 cannot by itself explain the loss of AQP4 that we observed. Normal-IgG, which does not include antibodies against AQP4, is also deposited around the needle insertion site, so upregulation of AQP4 cannot explain the IgG-specific deposition. The precise mechanism leading to deposition of IgG in and around the needle stick remains unclear. However, there appears to be selectivity for IgG deposition since a dextran tracer did not replicate these patterns. The mechanistic basis for this selectivity is unknown but may reflect Fc receptors or other Ig-binding structures in the CNS [28, 29].

This study confirms our previous finding that both NMO-IgG and normal-IgG diffuse from the CSF into the parenchyma after intrathecal injection via cisterna magna [14]. The low-level parenchymal IgG deposition bordering the CSF distal from the needle insertion that we observed after intrathecal injection is consistent with our previous study showing parenchymal IgG deposition 2 days post intrathecal injection [14]. Differences between the studies may reflect that whereas the localization of pathology and antibody distribution following intrathecal injection in our previous work [14] reflected three different deposition patterns, in the current study, a robustly predictable focus was created experimentally. Another study found that both NMO-IgG and normal-IgG were deposited in CNS parenchyma of rats when antibodies were continuously infused into the right ventricle using an osmotic mini pump [30]. In contrast, others found that when antibodies were infused into the intrathecal space in the spinal cord of rats and mice, only NMO-IgG infusion resulted in parenchymal deposition [31, 32].

Our observations suggest that IgG in CSF is distributed selectively from subarachnoid spaces to brain parenchyma at the site of an injury. We speculate that regional predilection of NMO lesions may reflect a combination of constitutive microenvironmental effects and external influences such as local infection which may also contribute to the localization of IgG deposition. The needle insertion that we have used is an experimental approach to model this hypothesis. Once the mechanism is established for IgG localization using experimental systems such as the one we describe, then the role of these different influences can be investigated.

## Abbreviations

AQP4: Aquaporin-4
BBB: Blood-brain barrier
CNS: Central nervous system
CSF: Cerebrospinal fluid
GFAP: Glial fibrillary acidic protein
HRP: Horseradish peroxide
IFNγ: Interferon gamma
IgG: Immunoglobulin-G
IL: Interleukin
ISF: Interstitial fluid
MS: Multiple sclerosis
NMO-IgG: Antibodies from NMO patients
NMOSD: Neuromyelitis optica spectrum disease
PBST: PBS containing 0.2% Triton X-100
TGFβ: Transforming growth factor beta
TNFα: Tumor necrosis factor alpha
